# Genetic mechanisms and age-related macular degeneration: common variants, rare variants, copy number variations, epigenetics, and mitochondrial genetics

**DOI:** 10.1186/1479-7364-6-13

**Published:** 2012-08-31

**Authors:** Melissa M Liu, Chi-Chao Chan, Jingsheng Tuo

**Affiliations:** 1 Laboratory of Immunology, National Eye Institute, National Institutes of Health, 10/10 N103, 10 Center Dr., Bethesda, MD 20892-1857, USA; 2 Johns Hopkins University School of Medicine, Baltimore, MD, 21205-2196, USA

**Keywords:** Age-related macular degeneration, Copy number variation, Genetics, Epigenetics, Mitochondria

## Abstract

Age-related macular degeneration (AMD) is a complex and multifaceted disease involving contributions from both genetic and environmental influences. Previous work exploring the genetic contributions of AMD has implicated numerous genomic regions and a variety of candidate genes as modulators of AMD susceptibility. Nevertheless, much of this work has revolved around single-nucleotide polymorphisms (SNPs), and it is apparent that a significant portion of the heritability of AMD cannot be explained through these mechanisms. In this review, we consider the role of common variants, rare variants, copy number variations, epigenetics, microRNAs, and mitochondrial genetics in AMD. Copy number variations in regulators of complement activation genes (*CFHR1* and *CFHR3*) and glutathione S transferase genes (*GSTM1* and *GSTT1*) have been associated with AMD, and several additional loci have been identified as regions of potential interest but require further evaluation. MicroRNA dysregulation has been linked to the retinal pigment epithelium degeneration in geographic atrophy, ocular neovascularization, and oxidative stress, all of which are hallmarks in the pathogenesis of AMD. Certain mitochondrial DNA haplogroups and SNPs in mitochondrially encoded NADH dehydrogenase genes have also been associated with AMD. The role of these additional mechanisms remains only partly understood, but the importance of their further investigation is clear to elucidate more completely the genetic basis of AMD.

##  

Age-related macular degeneration (AMD) is the leading cause of irreversible central vision loss in elderly populations in developed countries, and 30–50 million people are affected worldwide [1]. In the USA, it has been estimated that the prevalence of AMD is 13.4% in persons aged 60 years and older [2]. AMD primarily affects the photoreceptors, retinal pigment epithelium (RPE), Bruch's membrane, and choriocapillaris in the macula, the part of the retina responsible for central vision. It is classically characterized by the development of drusen, pathological extracellular deposits primarily containing glycolipids, proteins, and cellular debris, between the RPE and Bruch's membrane [3]. Small hard drusen can develop with normal aging. Early AMD occurs with more numerous and larger soft drusen in the macula, pigmentary changes in the RPE, and thickening of Bruch's membrane. Advanced AMD can manifest as either geographic atrophy (dry) or neovascular/exudative (wet) AMD. Geographic atrophy, or atrophy of the RPE, and degeneration of the overlying photoreceptors occurs in dry AMD. Wet AMD is characterized by the presence of choroidal neovascularization, leading to fluid leakage, hemorrhage, and disciform scar formation [4].

AMD is a complex and multifaceted disease involving contributions from both genetic and environmental influences. Epidemiological studies have identified age, gender, race, cigarette smoking, diet, and various cardiovascular risk factors as potential modulators of AMD risk, though age and cigarette smoking have been linked most consistently [5]. Numerous genomic regions and a variety of candidate genes have also been shown to impact AMD susceptibility. Although strong associations with AMD have been discovered, it is apparent that a significant portion of the heritability of AMD cannot be explained through these known mechanisms. In a recent meta-analysis of genome-wide association studies (GWAS) for advanced AMD, it was estimated that currently identified loci account for approximately 55% of the heritability of advanced AMD [6]. There is indeed a need to explore additional factors to elucidate the remaining genetic contributions to AMD susceptibility. In this review, we consider the role of common variants, rare variants, copy number variations, epigenetics, microRNAs, and mitochondrial genetics in AMD.

## Common variants

Linkage and association studies have implicated genetic modulators of AMD risk related to many mechanistic pathways, including oxidative stress, complement system dysregulation, DNA repair, mitochondrial dysfunction, neovascularization, and microglial recruitment [7]. Products of the complement system are present in drusen, and genetic variations in a number of complement components, including C2, C3, complement factor H (CFH), factor B, and factor I, have been linked to AMD [8]. Much of this work has revolved around single-nucleotide polymorphisms (SNPs). The role of SNPs in AMD has been extensively reviewed [9-12]. The Y402H polymorphism in *CFH* has been consistently demonstrated as a significant risk factor for AMD [13-15], and it has been estimated that the population attributable risk is between 47% and 69% [16]. SNPs in genes encoding other inflammatory markers such as *CX3CR1*[17] have also been associated with AMD. These findings support a central role for complement activation and inflammation in AMD pathogenesis. SNPs in age-related maculopathy susceptibility 2 (*ARMS2*) [18-20] and HtrA serine peptidase 1 (*HTRA1*) [21,22], two genes in strong linkage disequilibrium on chromosome 10q26 related to extracellular matrix function, are also associated with AMD susceptibility. Apoliproprotein E (apoE) polymorphisms modulate AMD risk as well, with the ε4 allelic variant conferring protection against AMD and the ε2 allelic variant increasing AMD risk [23]. Although controversial, AMD-associated SNPs have also been identified in excision repair cross-complementing group 6 (*ERCC6*), a DNA repair gene [24]; serpin peptidase inhibitor, clade G (C1 inhibitor), member 1 (*SERPING1*), an inhibitor of the complement component C1 [25]; and toll-like receptor 3 (*TLR3*) [26] and toll-like receptor 4 (*TLR4*) [27], mediators of the innate immune response.

Several recent GWAS studies have discovered novel AMD susceptibility loci. In a British population, the *TNXB-FKBPL-NOTCH4* region was identified [28]. The *TNFRSF10A-LOC389641* and *REST-C4orf14-POLR2B-IGFBP7* loci have also been identified as susceptibility loci for neovascular AMD in a Japanese population [29]. Variants in *SKIV2L*, which functions in exosome-mediated RNA degradation, and *MYRIP*, which plays a role in melanosome trafficking in the RPE, have been associated with AMD [30]. SNPs in the FRK/COL10A1 locus, which encodes the type X collagen alpha chain, and vascular endothelial growth factor A (*VEGFA*), a key regulator of neovascularization [31], have been associated with AMD as well [6], reiterating the role of extracellular matrix dysfunction and aberrant angiogenesis in AMD pathogenesis. A SNP in tissue inhibitor of metalloproteinases 3 (*TIMP3*), an inhibitor of extracellular matrix degradation, has been associated with AMD [32]. Hepatic lipase gene (*LIPC*) [33], cholesterylester transfer protein (*CETP*), lipoprotein lipase (*LPL*), and ATP-binding cassette, sub-family A, member 1 (*ABCA1*), all of which are associated with high-density lipoprotein metabolism, were also identified in this same GWAS study, suggesting that lipid and cholesterol dysregulation may be relevant in AMD as well.

## Rare variants

The concept that rare variants may contribute to the heritability of common diseases is well established. In 2010, a simulation by Sobrin et al. [34] demonstrated that common SNPs are insufficient to account for the AMD burden in densely affected families and that rare penetrant mutations may play a more important role. Several rare variants contributing to AMD risk have been identified. The rare H5 *CFH* haplotype, which includes a missense mutation leading to a R1210C substitution and functionally deficient CFH, has been identified as a causative variant in certain AMD patients [35]. This example demonstrates that both common and rare mutations in the same gene may modulate AMD risk. Missense mutations in the *FIBULIN-5* gene have also been found in 1.7% of patients in an American AMD population [36]. Rare variants in *HEMICENTIN-1* (*FIBULIN-6*), which encodes an extracellular matrix protein, have also been associated with AMD [37].

## Copy number variation

Copy number variation (CNV) is a common genetic structural variation that contributes greatly to human genetic diversity. CNVs are defined as deletions and duplications of genomic DNA segments that are at least one kilobase long [38]. Thousands of CNV loci have been identified throughout the human genome [39]. Several mechanisms have been proposed for the origination of CNV, including nonallelic homologous recombination, nonhomologous end-joining, and fork stalling and template switching [40]. The genomic rearrangements responsible for CNVs can lead to pathogenic phenotypes by modulating gene dosage, interrupting a gene, creating a fusion gene, exerting position effects, or unmasking a deleterious recessive mutation [41]. In fact, CNVs in specific genes have been linked to a variety of diseases, including Parkinson's disease, HIV infection, psoriasis, systemic lupus erythematosus, and a variety of neuropsychiatric diseases [42-46]. Nevertheless, the role of CNV in AMD remains incompletely understood.

It has been reported that deletions in *CFH*-related proteins 1 and 3 (*CFHR1* and *CFHR3*) are protective against AMD. *CFH* and *CFHR1–5*, regulators of complement activation, are located in a common locus in linkage disequilibrium on the long arm of chromosome 1. Hageman et al. identified a large deletion spanning *CFHR1* and *CFHR3* and demonstrated that homozygosity for this deletion conferred a protective effect, which was particularly evident for advanced AMD [47]. Hughes et al. identified this same deletion in two independent cohorts and demonstrated that its protective effects could not be attributed to the *CFH* Y402H genotype [48]. It was later demonstrated that its effects are also independent of the risk-modulating SNP in the CFH promoter region [49]. A previously characterized *CFH* haplotype, which is protective against AMD, occurs frequently in homozygotes for the *CFHR1* and *CFHR3* deletion. This copy number polymorphism may indeed be responsible for at least part of the protection conferred by the haplotype [50]. Schmid-Kubista et al. characterized CNV in *CFHR1* and *CFHR3* in 252 AMD patients and 249 controls using multiplex ligation-dependent probe amplification, which allows for quantitative determination of gene copy number [51]. This study reported novel CNVs in this locus, including homozygous deletions of only *CFHR1* or only *CFHR3*, heterozygous deletion of *CFHR3* only, and duplication of *CFHR1* only. Homozygous deletion of both genes decreased the odds of AMD substantially, and the protective effects of these CNVs were statistically significant for early AMD and neovascular AMD. The effects of heterozygous deletion did not achieve statistical significance, but a trend suggested a gene dosage effect. It was recently estimated that having fewer than two copies of *CFHR1* and *CFHR3* reduced the odds of having AMD by 43% [52]. *CFHR1* and *CFHR3* are regulators of the complement system that inhibit C5 convertase and C3 convertase, respectively. They are also competitive inhibitors of CFH and interrupt the binding of CFH to C3 [49]. A recent report suggests that the protection conferred by deletion of *CFHR1* and *CFHR3* may not be independent of rs1329428 and rs203687, two SNPs downstream of *CFH* Y402H in a novel 32-kb region associated with AMD risk [53]. This study also showed that a different deletion CNV in a 122-kb region encompassing *CFHR1* and *CFHR4* was associated with protection against AMD, independently of other CFH SNPs. The demonstrated role of CNVs and SNPs in modulating the activity of genes in the regulator of complement activation locus further highlights the mechanistic importance of the complement system in AMD pathogenesis.

CNVs in the glutathione S transferase (GST) genes are common in human populations, and their role in a variety of diseases has been investigated. GSTs are phase II enzymes that defend against oxidative stress and detoxify a variety of electrophilic compounds by covalent conjugation with glutathione [54]. Homozygous deletion of *GSTM1* and *GSTT1* occurs in approximately 50% and 20% of Caucasian individuals, respectively [55]. A decrease in *GSTT1* copy number has been linked with cortical cataract [56], and deletions of *GSTM1* and *GSTT1* are associated with asthma [57]. Preliminary investigations have been performed to evaluate the potential role of GST CNV in AMD. In a study by Oz et al., copy number genotyping was performed for *GSTM1*, *GSTT1*, and *GSTP1* in 35 patients with neovascular AMD and 159 controls, and no statistically significant associations were observed between *GSTM1*, *GSTT1*, or *GSTP1* individually and AMD [58]. However, homozygous deletions for *GSTM1/GSTT1* and *GSTM1/GSTP1* in combination were both found to significantly increase AMD risk. Guven et al. evaluated *GSTM1* and *GSTT1* CNV in 120 AMD patients and 198 controls in a Turkish population [59]. Homozygous deletion of *GSTM1* was found to be associated with AMD, and the association persisted for dry AMD after stratification by AMD subtype. Statistically significant associations were not observed between *GSTM1* or *GSTT1* genotype and neovascular AMD. A study by Kimura et al. in a Japanese population also reported no significant association between CNV in *GSTM1* or *GSTT1* and neovascular AMD [60].

The potential role for CNV in AMD has been evaluated for a number of other genes. A recent genome-wide scan for CNVs was performed using the Affymetrix GeneChip SNP Microarray in 400 AMD patients and 500 elderly controls. Nephrocystin 1 (*NPHP1*) and EGF containing fibulin-like extracellular matrix protein (*EFEMP1*) were identified as genes of interest [61]. Mutations in the *NPHP1* gene have been reported in Senior-Loken syndrome, which occurs with Leber congenital amaurosis, in Joubert syndrome, which may cause retinal dystrophy, and also in juvenile nephronophthisis in isolation. A deletion on chromosome 2q13 containing *NPHP1*, which functions in signal transduction and cytoskeleton organization, was observed in AMD patients, but not in controls. Duplication of a region upstream of *EFEMP1*, a gene that is mutated in Doyne honeycomb retinal dystrophy, was also observed in AMD patients, but not in controls. The reported CNVs were quite rare, and their mechanistic implications in the context of AMD remain entirely unknown and require further investigation.

We also recently evaluated the potential role of CNV in neovascular AMD for several candidate genes, including *CCR3*, *CFH*, *CX3CR1*, *ERCC6*, *HTRA1*, *VEGF*, *GSTM1*, and *GSTT1*[62]. Quantitative copy number genotyping was performed for each gene in 131 neovascular AMD patients and 103 elderly controls. Previously unreported CNVs were discovered in *CCR3*, *CX3CR1*, and *ERCC6*, but after adjustment for age, no statistically significant associations were found between CNVs in any of the evaluated genes and AMD. A trend in the unadjusted data suggested that an increase in *CX3CR1* copy number might be protective against AMD. This finding complements previously reported studies characterizing the association between two loss-of-function SNPs in *CX3CR1*, V249I and T280M [17], and elevated AMD risk. Additionally, histological studies have demonstrated that macular CX3CR1 protein levels are decreased in patients with AMD [63]. Further evaluation of the role of CNV in AMD is warranted to better understand the contribution of this type of genetic variation to AMD risk and pathogenesis.

## Epigenetics

Epigenetic modifications, or covalent modifications of genomic DNA that affect gene expression while preserving the DNA sequence, have also been investigated as potential genetic modulators of human disease. Methylation at the C5 position of cytosine is a common epigenetic modification that decreases gene transcription, and cytosine methylation in CpG dinucleotides can occur in clusters throughout the genome known as CpG islands. Covalent histone modifications, including methylation, acetylation, sumoylation, and phosphorylation, are also epigenetic modulators that affect gene transcription. Aberrant methylation patterns are associated with genomic instability and recognized as early genetic changes in certain tumors [64]. Loss of imprinting, or the epigenetic modifications that are inherited from parental chromosomes, has also been implicated in several conditions, including Beckwith-Wiedemann syndrome and Prader-Willi syndrome, and DNA methylation defects in T cells may contribute to systemic lupus erythematosus [65]. In experimental models, epigenetic patterns have also been shown to change with parental or embryonic exposure to dietary and environmental factors [66]. Global levels of genomic DNA methylation also decrease with age [67].

Based on these findings, epigenetic modifications may indeed serve as a genetic mechanism by which aging and environmental exposures modulate disease risk. These effects may be particularly pronounced in complex diseases of aging with demonstrated environmental contributions, such as AMD. The study of the role of epigenetics in AMD pathogenesis has only recently begun. A DNA methylation analysis was recently performed in three pairs of monozygotic twins with disparate AMD phenotypes to scan for genome-wide differences in methylation patterns. There were 256 genes with hypomethylated CpG sites and 744 genes with hypermethylated CpG sites in the twins with AMD (L Wei, unpublished work). The authors recently identified the *IL-17 RC* promoter, which is hypomethylated in AMD patients, as a locus of interest. Hypomethylation at this locus results in elevated expression levels of IL-17 RC in the peripheral blood cells and macular lesions of AMD patients (L Wei, unpublished work). The mechanistic implications of these differences require further investigation.

It is clear that environmental and dietary factors have a significant impact on AMD risk and progression. The Age-Related Eye Disease Study (AREDS) demonstrated that dietary supplements containing antioxidants such as zinc, beta-carotene, vitamin C, and vitamin E helped to decrease the risk of progression from intermediate to advanced AMD by 25% over 5 years [68]. Preliminary studies evaluating lutein, zeaxanthin, omega-3 fatty acids, and B vitamins have suggested that these compounds may confer protective effects as well [69]. A case series of monozygotic twin pairs with disparate AMD phenotypes has also been reviewed to assess the impact of behavioral and nutritional factors on AMD [70]. Twins with more advanced AMD tended to be heavier smokers and consumed less dietary vitamin D, betaine, and methionine. The modulatory effects of smoking and antioxidant supplementation on AMD confirm the critical role of oxidative stress in AMD pathogenesis. Additional studies are needed to investigate whether DNA methylation changes or other epigenetic modifications play a contributory role as well, but chronic low levels of antioxidant micronutrients may lead to changes in DNA methylation, synthesis, and repair.

## MicroRNAs

MicroRNAs are 19–25-nucleotide single-stranded noncoding RNAs that post-transcriptionally downregulate gene expression by binding the 3^′^ untranslated region (3^′^-UTR) of target mRNAs and marking them for cleavage by the RNA-induced silencing complex. MicroRNA genes are transcribed and trimmed to form pre-microRNAs with characteristic hairpin structures. Pre-microRNAs are cleaved by Drosha, exported from the nucleus, and processed again by Dicer in the cytoplasm to yield mature microRNAs [71]. It has been predicted that 30% of human genes may be regulated by microRNAs [72]. The role of microRNAs has been demonstrated in several contexts, including cancer [73] and immune function [74], and it is being investigated in AMD as well [75].

A recent study has shown that the NF-κB-regulated microRNAs miR-9, miR-125b, miR-146a, and miR-155 are upregulated in both AMD and Alzheimer's disease and that miR-146a and miR-155 target the 3^′^-UTR of CFH, thereby downregulating CFH [76]. A reduction in the Dicer1 enzyme and concomitant rise in Alu RNA has also been linked to the RPE degeneration in AMD patients with geographic atrophy [77]. Some studies have investigated the role of microRNAs in ocular neovascularization. It has been shown that microRNAs miR-31, miR-150, and miR-184 are significantly reduced in an ischemia-induced mouse model of retinal neovascularization and in a laser-induced mouse model of choroidal neovascularization in the absence of ischemia [78]. Intraocular injection with pre-miR-31 or pre-miR-150 significantly reduced the size of choroidal neovascular lesions. A recent study also demonstrated that knockdown of miR-23 and miR-27, which downregulate the antiangiogenic factors Sprouty2 and Sema6A, is protective against laser-induced choroidal neovascularization [79]. MicroRNA23a has also been found to be decreased in RPE cells from AMD donor eyes, and in ARPE-19 cells, it has been shown that antisense-mediated inhibition of miR-23a reduced cell growth and that the addition of a miR-23a mimic reduced H_2_O_2_-induced oxidative damage and Fas-mediated apoptosis [80]. As choroidal neovascularization and oxidative stress are hallmarks in AMD, these microRNAs may provide insights for the development of novel therapeutics.

## Mitochondrial genetics

Cells have several defense mechanisms against oxidative stress, including antioxidant compounds and enzymes and DNA repair machinery, but tissue damage can occur when these mechanisms are overwhelmed, and oxidative stress is believed to be a significant mechanistic contributor in AMD [81]. The retina is particularly susceptible to oxidative damage because it is among the most metabolically active tissues by weight in the body and because it is constantly under photochemical stress. Photoreceptors contain abundant mitochondria and produce high concentrations of reactive oxygen species (ROS) that when inadequately controlled lead to tissue damage, particularly in the underlying RPE. ROS can cause damage to macromolecules and organelles throughout the cell, but oxidative stress-induced mitochondrial DNA (mtDNA) damage has been especially studied as a pathogenic mechanism in AMD [82,83].

There are several factors that contribute to this phenomenon, including the localization of cell respiration reactions and ROS production in the mitochondria and decreased mtDNA repair mechanisms relative to nuclear DNA (nDNA) repair mechanisms, though some mtDNA damage can be corrected via base excision repair and mismatch repair [84]. The accumulation of mtDNA defects leads to an overall decline in mitochondrial function over time. In rodent RPE and choroid, the levels of the oxidative DNA damage marker 8-hydroxy-2^′^-deoxy-guanosine (8-OHdG) and the levels of deleted mtDNA increased with age, while the levels of certain DNA repair enzymes correspondingly decrease with age [85]. As a consequence of decreased mtDNA repair capacity, oxidative stress has been shown to preferentially damage mtDNA over nDNA in human RPE cells [86]. Accumulation of mtDNA deletions and cytochrome c oxidase deficiency has also been demonstrated with aging in human cone photoreceptors, particularly in the fovea [87]. The number of mtDNA rearrangements and deletions is also greater for the retina than for peripheral blood cells, in both AMD patients and age-matched normal controls [88], supporting the importance of the metabolically active retinal microenvironment and oxidative stress in mtDNA changes. In AMD retinas, mtDNA lesions are increased throughout the mitochondrial genome, and the degree of damage exceeds that which is seen in mtDNA with normal aging [89].

To more specifically characterize the mtDNA changes in AMD, several studies have compared mtDNA variations between AMD patients and normal controls. Jones et al. used restriction fragment length polymorphism analysis to determine mtDNA haplogroups for 3,302 Australian individuals from the Blue Mountains Eye Study, 317 of whom had either early or late AMD [90]. After adjusting for age, sex, and smoking status, mtDNA haplogroup H, the most prevalent haplogroup for European populations, was found to be protective against AMD, especially early AMD, and large soft drusen. Conversely, mtDNA haplogroup U was associated with increased RPE abnormalities. Udar et al. analyzed retinal mtDNA from the eyes of 11 AMD patients and 10 elderly controls and showed that AMD mtDNA had higher levels of oxidative damage, as evidenced by 8-OHdG staining, and a higher frequency of SNPs [91]. SNPs in the noncoding mtDNA control region at T16126C, T16126C + G13368A, A4917G + A73G, and T3197C +A12308G were strongly associated with AMD, and these SNPs correlated with mtDNA haplogroups J, T, and U. The A4917G SNP in the mitochondrially encoded NADH dehydrogenase gene, which defines mtDNA haplogroup T, was also shown in separate studies to be associated with increased risk of AMD, potentially by disrupting cell respiration and increasing the production of ROS [92,93]. Carrying the A11812G and A4917G SNPs, both of which lie in NADH ubiquinone oxidoreductase genes, has been associated with a 2.5-fold elevated risk of developing advanced AMD [93].

## Conclusions

AMD is a significant cause of irreversible blindness in the elderly, and the global burden of this disease is ever-growing as populations age. The genetic basis of this disease is only partially understood. However, it is hoped that further investigations may lead to the identification of novel therapeutic pathways. Technological advancements have facilitated the exploration of mitochondrial genetics, epigenetic modifications, and genetic structural variations such as CNV in not only AMD but also a variety of other common diseases. The role of CNV in AMD has thus far proven to be moderate, but the evidence suggests that further studies are warranted. The preliminary experiments evaluating epigenetic modifications in AMD require replication in independent study populations, and additionally, given the demonstrated importance of the retinal microenvironment in AMD, it may be illustrative to evaluate these types of genetic modifications in retinal tissues, not only in peripheral blood mononuclear cells. There is a wide array of heterogeneity in AMD phenotype and response to therapy, and a more complete understanding of the genetics of AMD is critical. Many SNPs have been reproducibly demonstrated in AMD risk and pathogenesis, but there are clearly additional genetic mechanisms that remain to be identified. Common variants explain approximately 55% of the heritability of AMD. Mechanisms such as rare variants, copy number variations, epigenetics, and mitochondrial inheritance may contribute to the missing heritability. Considering more fully the predisposing genetic factors in AMD may ultimately help to identify high risk populations, predict disease progression, and anticipate response to personalized therapy.

## Competing interests

The authors declare that they have no competing interests.

## Authors' contributions

MML drafted the manuscript. CCC provided critical review. JT determined the scope and focus of the manuscript and participated in its design and review. All authors read and approved the final manuscript.

## Author details

^1^Laboratory of Immunology, National Eye Institute, National Institutes of Health, 10/10 N103, 10 Center Dr., Bethesda, MD 20892-1857, USA. ^2^Johns Hopkins University School of Medicine, Baltimore, MD 21205-2196, USA.
